# Comparison of 4 different RNA sources from lactating dairy cows to assess the mammary transcript abundance

**DOI:** 10.3168/jdsc.2025-0768

**Published:** 2025-09-04

**Authors:** E.M. Shangraw, M.C. Lucy, T.B. McFadden

**Affiliations:** Division of Animal Sciences, University of Missouri, Columbia, MO 65211

## Abstract

Noninvasive sampling of milk fat for isolation of RNA is an alternative technique to assess the mammary transcriptome. However, contamination of RNA from milk fat with nonmammary sources of RNA may complicate the interpretation of results. We measured transcript abundance of 8 genes in RNA from milk fat, milk somatic cells, mammary tissue, and blood leukocytes to determine how the transcript abundance of selected genes compares between RNA sources. Samples from 8 healthy cows were harvested immediately after slaughter. Blood was collected during exsanguination. Milk and mammary tissue were collected from the same mammary gland. Mammary tissue was frozen immediately, and fluids were centrifuged to collect the buffy coat from blood, and the fat layer and cell pellet from milk. The RNA isolated from all tissue sources was prepared for reverse-transcription quantitative PCR. Relative transcript abundance for each gene was determined by normalizing the abundance of the target gene against the abundance of reference genes (*RPL4* and *RPS23*). Differences in relative transcript abundance were determined per gene by Friedman tests and per subsets of genes by correlations. For the selected 8 genes, correlations were modest and tended to show positive relationships between RNA from milk fat, milk somatic cells, and mammary tissue, depending on the function of the gene. The 4 milk-related genes encoding milk proteins (*CSN2*, *LALBA*) and enzymes involved in fat metabolism (*FASN*, *LPIN1*) were most highly expressed in RNA isolated from milk fat and mammary tissue. The remaining 4 genes (*ITGB2*, *CD68*, *NFKBIA*, and *HK1*) were related to immune function and were mainly expressed in RNA from milk somatic cells, with fewer transcripts from milk fat and few to no transcripts from mammary tissue or blood leukocytes. Relative transcript abundance in RNA from milk fat was comparable to transcript abundance in RNA from mammary tissue for milk-related genes, but for immune-related genes, it was more similar to RNA from milk somatic cells than from mammary tissue or blood leukocytes. Thus, transcript abundance of the assessed genes was uniquely controlled between each of the 4 tissue sources. Using RNA from milk fat may be beneficial for studies investigating lactational and immunological responses of the mammary gland based on its similar transcript abundance patterns compared with RNA from both mammary tissue and milk somatic cells.

## Highlights

• Milk-specific gene transcripts were most abundant in milk fat and mammary tissue RNA.
• Immune gene transcripts were most abundant in RNA from milk somatic cells.
• Transcript abundance from blood leukocyte RNA did not match that of milk somatic cells.

## Graphical Abstract

**Figure:**
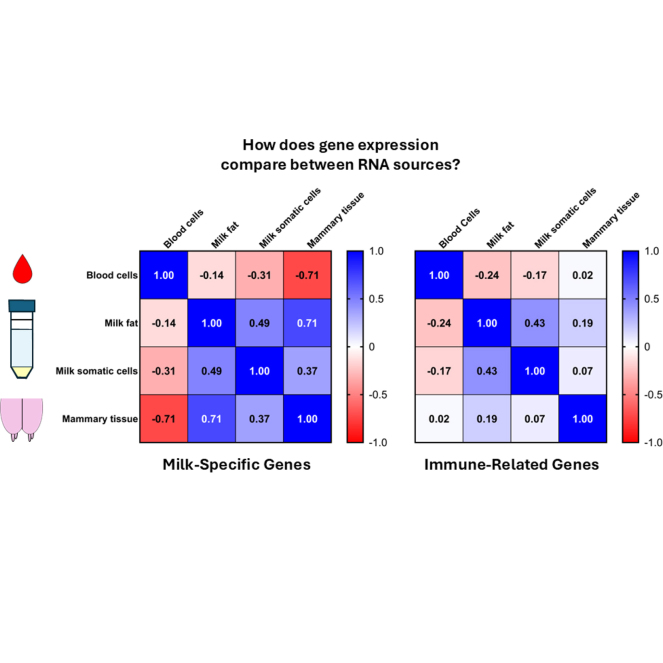
**Summary:** The mammary transcriptome, historically sourced from mammary tissue obtained by biopsies, may also be sourced from milk fat or milk somatic cells. We measured transcript abundance of selected genes in 2 of the common pathways assessed in lactation studies, milk synthesis and immune responses, to compare transcript abundance in RNA from 4 different tissue sources: blood leukocytes, milk fat, milk somatic cells, and mammary tissue. Whereas relative transcript abundance in RNA from blood leukocytes was not similar to milk somatic cells, transcript abundance in RNA from milk fat modestly correlated with both mammary tissue and milk somatic cells, depending on whether the genes were milk- or immune-related, respectively. Sampling RNA from milk fat rather than RNA from mammary tissue appears to be useful for studies investigating both lactational and immunological responses.

Isolating RNA from milk fat is one technique used to quantify mammary gland transcript abundance during lactation (Maningat et al., 2007; Brenaut et al., 2012; Lemay et al., 2013). Historically, gene expression studies investigated the mammary transcriptome using mammary tissue obtained by biopsy or at slaughter (Molenaar et al., 1992; Finucane et al., 2008). Other studies have used milk somatic cells (Boutinaud and Jammes, 2002; Boutinaud et al., 2013), which would allow for measurement of RNA in all the captured cells or selectively by cell type, such as exfoliated mammary epithelial cells (**MEC**) after antibody capture. The technique of isolating RNA from milk fat assumes that milk fat globules capture RNA within cytoplasmic crescents when milk fat globules are secreted by MEC (Heid and Keenan, 2005; Lemay et al., 2013). This biological process allows researchers to assess the mammary transcriptome without resorting to invasive sampling of mammary tissue. However, whether RNA isolated from milk fat represents RNA isolated from mammary parenchymal tissue, specifically from the MEC, is debatable and may be skewed by several factors, including degradation of RNA in milk fat (Lemay et al., 2013) or inclusion of milk somatic cells that express nonmammary transcripts (Brenaut et al., 2012; Shangraw and McFadden, 2024). Previously, one study compared the transcript abundance of 5 sources of bovine RNA (mammary biopsy, milk fat, laser-captured MEC, total milk somatic cells, and antibody-captured milk MEC isolated from somatic cells) and determined that of all the sources, RNA from milk fat was most closely correlated with both total milk somatic cells and mammary tissue (Cánovas et al., 2014). The study involved a limited number of cows (n = 3). Additionally, no comparison was made to RNA from circulating leukocytes, a separate pool of immune cells from which most milk somatic cells are derived (Paape et al., 2003). This is intriguing because MEC typically represent <1% to 3% of all milk somatic cells (Boutinaud and Jammes, 2002; Schwarz et al., 2011), yet Cánovas et al. (2014) found transcript abundance in RNA from mammary biopsy was closer to RNA from milk somatic cells than to antibody-captured milk MEC. Thus, the objective of this study was to confirm that the relative transcript abundance of RNA from milk fat is representative of the abundance of RNA from mammary tissue and to extend our knowledge of transcript abundance to RNA from blood leukocytes. We hypothesized that transcript abundance of our chosen genes in milk fat would be more highly correlated with mammary tissue than milk somatic cells, the latter of which would be more correlated with blood leukocytes.

Samples were collected from Holstein cows (n = 8) immediately after slaughter. Cows were milked ∼24 h before collection. Cows were slaughtered by captive bolt stunning and exsanguination at the university abattoir. Blood was collected during exsanguination into tubes containing 18 mg EDTA. Udders were removed from the carcass 15 to 20 min after stunning. The right rear teat was identified, cleaned of any debris, and 30 to 40 mL of milk stripped into a sterile 50-mL conical tube. There was no evidence of clinical mastitis in milk samples. The right rear quarter was then split along the caudal aspect until halfway between the dorsal surface and the teat. A 1 × 1 × 0.5-cm sample of mammary tissue was dissected 5 to 7 cm from the outer skin to replicate mammary biopsy collection, then placed in a cryovial and immediately frozen in liquid nitrogen. Blood and milk samples were kept on ice after collection for <30 min.

Blood and milk samples were centrifuged at 2,000 × *g* for 30 min at 4°C. One milk sample had a visually larger cell pellet than other samples after centrifuging. One gram of milk fat was transferred into 3 mL of Trizol (Invitrogen, Life Technologies), and then the remaining fat and skim milk were drained to allow collection of the milk somatic cells by resuspending in 1 mL of Trizol. For blood leukocyte collection, excess plasma was pipetted off, and then the buffy coat was transferred with minimal red blood cells into 1 mL of Trizol. All samples were vortexed vigorously immediately after transfer into Trizol. The RNA from milk fat and milk somatic cells was isolated as described previously (Shangraw and McFadden, 2024). The RNA from buffy coats was isolated by centrifuging samples in Trizol at 17,000 × *g* for 3 min at 4°C, then transferring the supernatant to a gDNA column for clean-up. After centrifuging the gDNA columns at 17,000 × *g* for 30 s, the flow-through was used for the 2-step chloroform extraction and column purification procedure used for milk samples. Lastly, 10 to 12 mg of frozen mammary tissue per cow was homogenized to isolate RNA using the RNeasy Plus Mini kit (Qiagen). All RNA was treated on-column with 10 µL of DNase I in 70 µL of buffer, followed by wash steps. The RNA was eluted from the final column with 30 µL of nuclease-free water and stored at −80°C.

The RNA concentration was determined by NanoDrop (Thermo Scientific). The ratio of absorbance at 260/280 nm averaged 2.03 ± 0.07 for all samples. Quality was assessed for all samples by gel electrophoresis. The RNA from blood leukocytes showed distinct 28S and 18S bands, whereas these bands were fainter in RNA from mammary tissue and milk somatic cells. The RNA from milk fat contained little to no rRNA bands and mainly lower molecular weight bands, as expected (Brenaut et al., 2012; Cánovas et al., 2014). For cDNA construction, each reaction contained 1 µg of RNA per sample. Each 20-µL reaction contained 10 µL of sample and 10 µL of master mix (High Capacity cDNA, Applied Biosystems): 2 µL of 10× buffer, 0.8 µL of 25× dNTP, 2 µL of random primers, 1 µL of MultiScribe reverse transcriptase (Invitrogen), and 4.2 µL of nuclease-free water. The reaction was performed in a T100 thermal cycler (BioRad Laboratories Inc.), programmed to 25°C for 10 min, 37°C for 120 min, and 85°C for 5 min, then held at 4°C. After synthesis, 5 µL was pipetted from each sample to create a cDNA pool and 10 µL of cDNA from the remaining sample was diluted 1:10 with RNase-free water for storage at −20°C.

Primer sequences for the selected genes (*CSN2*, *LALBA*, *FASN*, *LPIN1*, *ITGB2*, *CD68*, *NFKBIA*, *HK1*, *RPL4*, *RPS23*) in the current study were reported previously (Shangraw and McFadden, 2024). Primer efficiency was validated using a 5-point dilution series (from 1.25 ng to 6.67 pg of reverse-transcribed RNA) of pooled cDNA from all samples and a no-template control. The same dilution series used to validate primer efficiency was used as the standard curve for quantitative PCR (**qPCR**).

For real time qPCR, samples and the standard curve were plated in duplicate in 384-well plates, with each gene run on a single plate. Each amplification reaction contained 5 µL of SYBR Green mix (PerfeCTa SYBR Green SuperMix, Quantabio) 2 µL of a 50:50 mix of forward and reverse primers with an optimized final primer concentration of 200 to 800 n*M*, 1 µL of water, and 2 µL of standard or sample. Plates were run in a C1000 Touch thermal cycler (BioRad Laboratories Inc.) using the following cycling protocol: polymerase activation at 95°C for 3 min, then 40 cycles at 95°C for 15 s (denaturation), 60°C for 20 s (annealing), and 68°C for 20 s (extension), followed by a melting curve from 65°C to 95°C using an incremental temperature increase of 0.5°C every 10 s. Primer efficiencies ranged from 0.85 to 1.03, mean 0.93 ± 0.02, with R^2^ ≥ 0.99. Quantification cycle (**C_q_**) values were imported into Microsoft Excel for further analyses. Arbitrary cDNA amounts were determined from the C_q_ and the slope of the standard curve by the following equation: 10^[(Cq – b)/m]^, where b = intercept and m = slope. The geometric mean of the 2 internal control genes (*RPL4* and *RPS23*) was used to normalize transcript abundance of all target genes. Four genes (*RPL4*, *RPS23*, *HK1*, and *NFKBIA*) were tested as possible reference genes using BestKeeper (Pfaffl et al., 2004). The least stable, *NFKBIA*, was eliminated. Standard deviations (±C_q_) for *RPL4*, *RPS23*, and *HK1* were 1.35, 1.36, and 1.39, respectively. The combination of all 3 genes gave an SD of 1.14, slightly more stable than *RPL4* and *RPS23* combined (SD = 1.24), but we chose to use the latter 2 because the pattern of *HK1* abundance across tissues was remarkably similar to that of the immune-related genes. The same was true of *NFKBIA*, so both were included as immune-related target genes.

Statistical comparisons were run using GraphPad Prism 10.2.3 (GraphPad Software). For *CSN2* and *LALBA*, cows (n = 2) with a missing arbitrary amount measured from blood leukocytes were removed from the test, one of which had the milk sample with a large cell pellet. Nonparametric tests were used for correlations (Spearman) and pairwise comparisons (Friedman test). Correlation analyses were run for all genes and also split between genes with functions related to milk synthesis (*CSN2*, *LALBA*, *FASN*, *LPIN1*) or the immune system (*ITGB2*, *CD68*, *NFKBIA*, *HK1*). Significance was declared at *P* ≤ 0.05 and tendency at *P* ≤ 0.10.

Although they were acceptably stable for use in normalization, the mean transcript abundance of the 2 reference genes differed between sample types. Blood leukocytes expressed 16.4 times more transcripts of the reference genes than milk fat (*P* < 0.05). Similarly, mammary tissue and milk somatic cells expressed 5.5 and 5.0 times more reference gene transcripts compared with milk fat, respectively, though only mammary tissue was significantly different (*P* < 0.05). This result suggested that, like rRNA, there is selection against sequestration of ribosomal protein transcripts (e.g., *RPL4* and *RPS23*) into milk fat globules. Whether some transcripts are selected for and disproportionately abundant in RNA from milk fat globules is unknown. However, RNA isolated from bovine whey also included little to no rRNA but a relatively high abundance of milk protein transcripts (Izumi et al., 2012).

The correlations for relative transcript abundance between different tissue sources depended on which genes and which cows were included in the analysis (Table 1). When all genes and all cows were included, the only significant correlation was between RNA from milk somatic cells and mammary tissue (Spearman's rho = 0.76, *P* = 0.04). However, when the 2 cows with missing data from blood leukocytes were removed from the analysis, the strongest correlation was between RNA from milk fat and mammary tissue (Spearman's rho = 0.77, *P* = 0.10). Grouping our genes into milk-related (*CSN2*, *LALBA*, *FASN*, *LPIN1*) and immune-related genes (*ITGB2*, *CD68*, *NFKBIA*, *HK1*) revealed only 2 significant positive correlations for either set of cows. Although all correlations for milk-related genes were not different, we noted some modest correlation coefficients. As with all genes, the strongest correlation for milk-related transcript abundance when all cows were included was between RNA from milk somatic cells and mammary tissue (Spearman's rho = 0.69, *P* = 0.07), but the strongest correlations when cows with missing data were removed were between RNA from milk fat and mammary tissue (Spearman's rho = 0.71, *P* = 0.14) in addition to RNA from blood leukocytes and mammary tissue (Spearman's rho = −0.71, *P* = 0.14). For immune-related transcript abundance, there were no correlations between any tissue sources when all cows were included, but with the smaller set of cows, there was 1 positive correlation between RNA from blood leukocytes and mammary tissue (Spearman's rho = 0.89, *P* = 0.03). Both sets of cows also showed a modest positive correlation for immune-related transcript abundance between RNA from milk fat and milk somatic cells. To interpret these correlations, we looked more closely at the relative transcript abundance of individual genes between RNA sources.

**Table 1:** Transcript abundance correlations between tissue sources, gene function, and cows

Source 1	Source 2	All genes		Milk genes^1^		Immune genes^2^	
		n = 6^3^	n = 8^4^	n = 6^3^	n = 8^4^	n = 6^3^	n = 8^4^
Blood cells	Milk fat	−0.71	−0.45	−0.14	−0.55	0.20	−0.24
Blood cells	Milk cells	−0.26	−0.38	−0.31	−0.10	−0.14	−0.17
Blood cells	Mammary tissue	−0.60	0.05	−0.71	−0.38	0.89*	0.02
Milk fat	Milk cells	0.37	0.45	0.49	0.41	0.66	0.43
Milk fat	Mammary tissue	0.77†	0.21	0.71	0.50	−0.20	0.19
Milk cells	Mammary tissue	0.71	0.76*	0.37	0.69†	−0.37	0.07

1 *CSN2*, *LALBA*, *FASN*, *LPIN1*.

2 *ITGB2*, *CD68*, *NFKBIA*, *HK1*.

3 Spearman correlation coefficient for 6 cows with no missing data.

4 Spearman correlation coefficient for all 8 cows in the study.

* *P* ≤ 0.05; †*P* ≤ 0.10.

Transcripts encoding the milk proteins β-casein (*CSN2*) and α-lactalbumin (*LALBA*) were most abundant in RNA from mammary tissue and were not different when comparing relative abundances in tissue against milk fat (Figure 1). A similar pattern was observed for the relative transcript abundance of genes encoding enzymes involved in fatty acid synthesis and metabolism, fatty acid synthase (*FASN*), and lipin 1 (*LPIN1*). It should be noted that our results likely underestimate transcript abundance of milk-related genes under normal 8- or 12-h milking intervals because our cows were not milked for 24 h before sample collection. Although abundance of milk-related gene transcripts remained relatively high in RNA from milk fat and mammary tissue, Singh et al. (2008) reported substantial reduction of *LALBA* and α- and κ-casein mRNA by 24 h after milking compared with 6 h. As expected, the relative transcript abundance of milk-related genes in RNA from blood leukocytes was negligible and significantly lower than from mammary tissue or milk fat (*P* < 0.05). Further, the abundance of these genes in RNA from milk somatic cells was not significantly different compared with either RNA from blood leukocytes or milk fat. Finally, the transcript abundance of *CSN2* and *FASN*, but not *LALBA* and *LPIN1*, was greater in RNA from mammary tissue than from milk somatic cells (*P* < 0.05). The phagocytosis of milk by milk somatic cells and the inclusion of some exfoliated MEC (Boutinaud and Jammes, 2002) likely explains why we measured more transcripts for these genes in milk somatic cells than in blood leukocytes, which can express low levels of milk-specific proteins (Tokarska et al., 2009).

**Figure 1:**
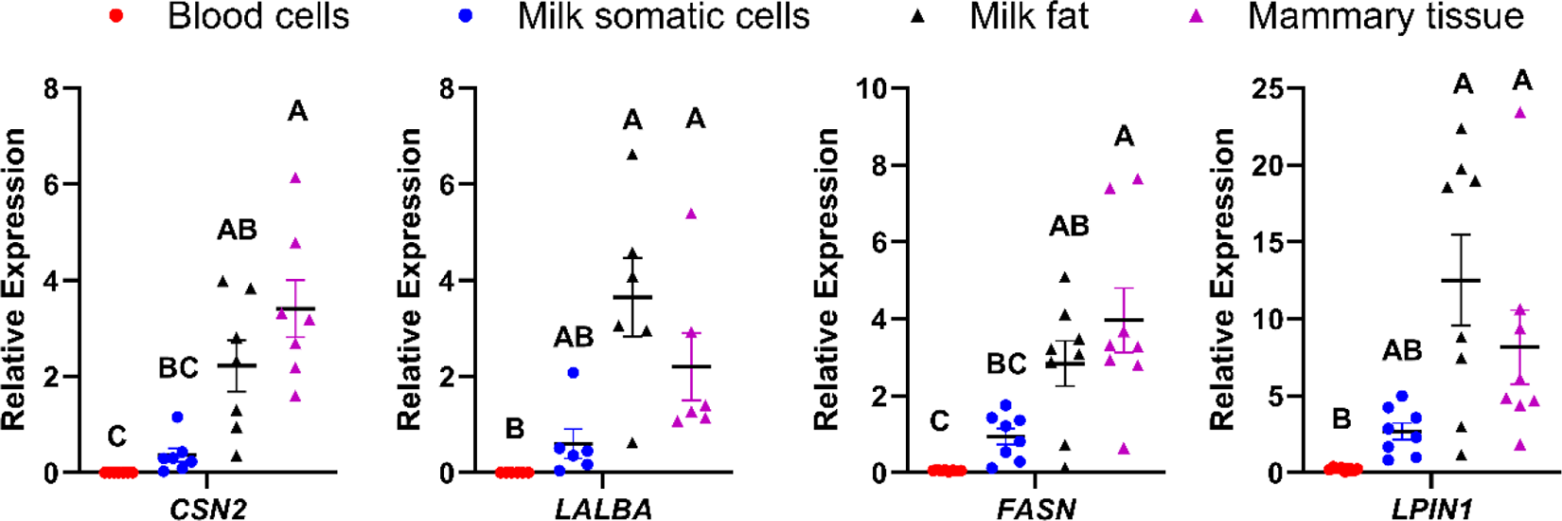
Normalized transcript abundance of genes related to milk synthesis. We isolated RNA from 4 tissue sources (blood cells, milk somatic cells, milk fat, and mammary tissue) from healthy Holstein cows. Each gene is expressed relative to the geometric mean transcript abundance of 2 reference genes in the same sample. Different letters (A–C) indicate a significant difference between tissue sources at *P* ≤ 0.05. Plots indicate mean ± SEM relative transcript abundance per tissue source.

The relative transcript abundance of the immune-related genes, *ITGB2*, *CD68*, *NFKBIA*, and *HK1*, was greatest in RNA from milk somatic cells, followed by RNA from milk fat, then RNA from blood leukocytes, and lowest in RNA from mammary tissue (Figure 2). Except for *ITGB2*, the relative transcript abundance in RNA from milk somatic cells was greater than in blood leukocytes or mammary tissue (*P* < 0.05). Only RNA from milk somatic cells contained greater amounts of *ITGB2* than RNA from mammary tissue (*P* < 0.001). The relative transcript abundance of *NFKBIA* in RNA from milk somatic cells was greater than in RNA from milk fat (*P* < 0.05). However, transcript abundance of all other immune-related genes in RNA from milk somatic cells was greater but not different from RNA from milk fat, partially due to the high variability in both tissue sources. Likewise, there was no difference in the relative transcript abundance of RNA from blood leukocytes compared with RNA from milk fat or mammary tissue. However, RNA from milk fat contained greater amounts of *CD68* and *HK1* than RNA from mammary tissue (*P* < 0.05).

**Figure 2:**
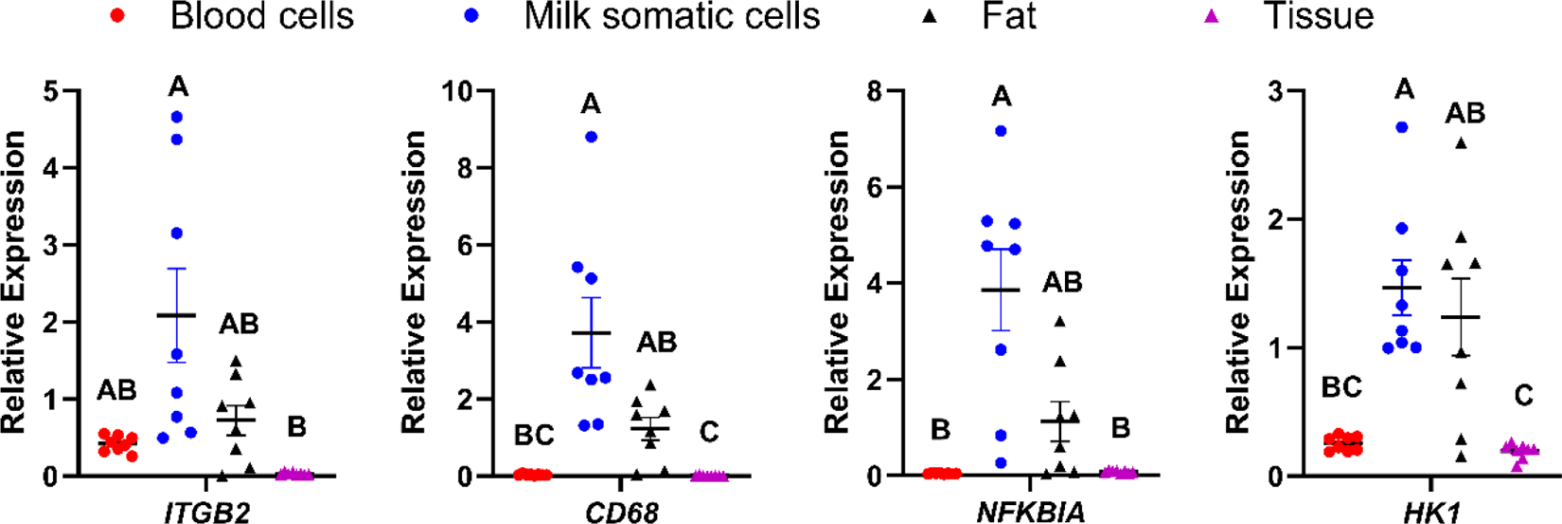
Normalized transcript abundance of genes related to immune response. We isolated RNA from 4 tissue sources (blood cells, milk somatic cells, milk fat, and mammary tissue) from healthy Holstein cows. Each gene is expressed relative to the geometric mean transcript abundance of 2 reference genes in the same sample. Different letters (A–C) indicate a significant difference between tissue sources at *P* ≤ 0.05. Plots indicate mean ± SEM relative transcript abundance per tissue source.

From a biological perspective, the difference between tissues for immune-related transcripts was reasonable. The similarity between milk cells and milk fat was unsurprising because both were isolated from the same milk samples. Milk somatic cells phagocytize milk within the gland (Paape et al., 2003), and milk somatic cells can be recovered from milk fat (Sarikaya, 2006). However, the marked difference between blood leukocytes and milk somatic cells was unexpected. To be isolated from milk, milk somatic cells must migrate from the circulation and extracellular space into the milk. Although the mechanisms controlling mammary recruitment of milk somatic cells in healthy, nonmastitic cows are still being identified, there is evidence for constitutive secretion of the chemokine CXCL3 into milk (Rainard et al., 2008). This chemokine and other bioactive molecules in milk likely alter transcription and immune function in blood-derived milk somatic cells. Our chosen immune-related genes and the cells present in the samples may also have influenced the results. The immune-related genes included 2 cell surface markers for leukocytes, an inhibitor of inflammation, and *HK1*, a key enzyme for glycolysis that is upregulated in myeloid cells during inflammation (Nishizawa et al., 2014). Whereas all our selected genes were more highly expressed in milk somatic cells than blood cells, Pfaffl et al. (2003) found the opposite for *ALOX5* transcript abundance. Different numbers and proportions of immune cell types could explain the variability both between and within tissue sources because milk samples with similar low cell counts can have markedly different cellular proportions (Schwarz et al., 2011). Total and differential cell counts were not measured in the current study, though we did note that 1 milk sample had a qualitatively large cell pellet. Despite these limitations, the transcript abundance of all 4 immune-related genes showed a consistent pattern between tissue sources.

None of the tissue sources selected for this study had a similar transcript abundance profile to another, though our analysis included only a relatively small number of genes. Previous studies have run transcriptomic analyses to compare tissue sources to assess whether one or more could be used in place of invasive mammary biopsies. Some concluded that milk fat was representative of mammary tissue (Brenaut et al., 2012; Cánovas et al., 2014) whereas others argued for the use of milk fat rather than mammary tissue because it was enriched for MEC-specific transcripts (Lemay et al., 2013). A global, unbiased assessment of transcript abundance using transcriptomes is ideal but could still result in nonsignificant differences when assessing individual genes and pathways. Brenaut et al. (2012) further qualified the use of milk fat as a substitute for mammary tissue, finding evidence of contamination of RNA from milk fat with RNA from immune cells during the later stages of an intramammary infection, but not the early stages. This, along with our immune-related transcript abundance data, suggests that RNA from milk fat may not be representative of RNA from mammary tissue under mastitic conditions or when the likelihood of milk somatic cell content is high. However, if the aim of the researcher is to assess both the mammary transcriptome and immune processes, milk fat could be a better source of RNA than either mammary tissue or milk somatic cells, given that RNA from milk fat is comparable to both tissue sources for different genes. Thus, care must be taken when choosing between mammary tissue, milk fat, or milk somatic cells to assess the mammary transcriptome.

In summary, we conclude that RNA from milk fat is not strictly representative of RNA from mammary tissue or milk somatic cells. When assessing individual milk-related genes among the 4 tissue sources, relative transcript abundance in RNA from milk fat was most comparable to RNA from mammary tissue. In contrast, immune-related genes were expressed more similarly in RNA from milk fat and milk somatic cells compared with mammary tissue. We further rejected our hypothesis that the transcript abundance of immune-related genes in RNA from milk somatic cells would be more highly correlated with RNA from blood leukocytes than the other tissue sources. The RNA from milk fat does not recapitulate relative transcript abundance in RNA from mammary tissue but may be an acceptable substitute to assess genes associated with lactational and immunological responses in the bovine mammary gland.
